# CaMKK-CaMK1a, a New Post-Traumatic Signalling Pathway Induced in Mouse Somatosensory Neurons

**DOI:** 10.1371/journal.pone.0097736

**Published:** 2014-05-19

**Authors:** Lucie Elzière, Chamroeun Sar, Stéphanie Ventéo, Steeve Bourane, Sylvie Puech, Corinne Sonrier, Hassan Boukhadaoui, Agnès Fichard, Alexandre Pattyn, Jean Valmier, Patrick Carroll, Ilana Méchaly

**Affiliations:** 1 Institute for Neurosciences of Montpellier, I.N.S.E.R.M. U1051, Montpellier, France; 2 Department BioMV, University of Montpellier II, Montpellier, France; 3 Molecular Neurobiology Laboratory, The Salk Institute, La Jolla, California, United States of America; University of Louisville, United States of America

## Abstract

Neurons innervating peripheral tissues display complex responses to peripheral nerve injury. These include the activation and suppression of a variety of signalling pathways that together influence regenerative growth and result in more or less successful functional recovery. However, these responses can be offset by pathological consequences including neuropathic pain. Calcium signalling plays a major role in the different steps occurring after nerve damage. As part of our studies to unravel the roles of injury-induced molecular changes in dorsal root ganglia (DRG) neurons during their regeneration, we show that the calcium calmodulin kinase CaMK1a is markedly induced in mouse DRG neurons in several models of mechanical peripheral nerve injury, but not by inflammation. Intrathecal injection of NRTN or GDNF significantly prevents the post-traumatic induction of CaMK1a suggesting that interruption of target derived factors might be a starter signal in this *de novo* induction. Inhibition of CaMK signalling in injured DRG neurons by pharmacological means or treatment with CaMK1a siRNA resulted in decreased velocity of neurite growth *in vitro*. Altogether, the results suggest that CaMK1a induction is part of the intrinsic regenerative response of DRG neurons to peripheral nerve injury, and is thus a potential target for therapeutic intervention to improve peripheral nerve regeneration.

## Introduction

Peripheral nerve damage leads to adaptive responses allowing injured neurons to survive and to re-grow to their targets. However, the regeneration process is slow and incomplete [1]. It is also often accompanied by disturbing motor, autonomic and sensory consequences including motor dysfunctions and neuropathic pain conditions that are difficult to treat. A better understanding of the molecular and cellular processes occurring in this pathology is important for designing effective therapies.

The molecular events underlying post-traumatic responses involve complex steps occurring in a temporal sequence and are age and type of injury dependant [2], [3]. They include rapid, mid- and long-term changes the significance of which in terms of pathological or regenerative processes have not yet been fully elucidated. A part of this response involves the reprogramming of developmental processes implicated in neurite outgrowth as well as the induction of a set of *de novo* expressed genes [4]–[6] some of which play roles in mature neuron re-growth [7], [8].

In transcriptomic analyses of axotomised sensory neurons from mouse DRG, we observed changes in the expression of a cluster of genes associated with calcium signalling [4]. Within this cluster, we found a striking elevation of the expression of Calcium/Calmodulin-dependant protein kinase 1 also known as Calcium/Calmodulin-dependant protein kinase 1 alpha (and hereafter referred to as *CaMK1a*) three days after sciatic nerve axotomy. The role of calcium signalling in neurite growth, dendrite morphology and axon pathfinding is well documented in many systems ([9]–[11] for reviews). Amongst the targets of calcium signalling are the CaM-dependant protein kinases; including myosin light chain kinase, phosphorylase kinase, EF-2 kinase (CaMKinase3) and the multi-functional enzymes, CaMKinases 1, 2 and 4. CaMK1s exist as multiple isoforms transcribed from the genes *CaMK1a*, *CaMK1b*, *CaMK1d* and *CaMK1g* [12]. They are abundantly expressed in the central nervous system throughout development with specific regional and sub-cellular distributions [12], and regulate a variety of processes such as dendrite growth and plasticity ([13] for review, [14]. However, the expression and specific role of CaMK1 family members in the peripheral nervous system remains unknown.

In this study, we show that up-regulation of *CaMK1a* expression is part of the response of adult DRG neurons specifically occurring after physical injury to the peripheral nerve. *CaMK1a* expression is indeed absent in naive DRG neurons and is not induced by CFA inflammation. When induced in injured sensory neurons, the protein is present in the cell bodies and along peripheral projections. GDNF family ligands delivery counteracts the *de novo* induction of CaMK1a expression, suggesting that CaMK1a expression is part of the transcriptional program induced by the interruption of retrograde signalling subsequent to the lesion. Moreover pharmacological inhibition of CaMKK or treatment of axotomized sensory neurons with CaMK1a siRNA reduced the velocity of neurite outgrowth of these neurons *in vitro*.

## Materials and Methods

### Animals and surgical procedures

Animals were housed in facilities accredited by the French ministry of agriculture and forestry,(B-34 172 36–March 11, 2010). Experiments were carried out in accordance with the animal welfare guidelines of the French medical research institute (INSERM) and with the European Communities Council Directive of 24 November 1986 (86/609/EEC) regarding the care and use of animals for experimental procedures, and were approved by our local Ethics committee for animal experiments, (Languedoc Roussillon, N° 34–376, February the 17th of 2009). All efforts were made to minimize the number and suffering of the animals used. Embryos (E13) and early post-natal, (P0) Swiss mice were killed by decapitation. Surgery was performed on adult Swiss mice (7–8 weeks old) deeply anaesthetized by isoflurane inhalation. The left sciatic nerve was exposed at the mid-thigh level and sectioned (3- to 5-mm fragment of nerve was removed) or compressed 15 sec with fine forceps (Moria n°5). A chronic constriction injury (CCI) was performed by a loose ligation of the sciatic nerve [15] and the Von Frey behavioural test was carried out to confirm the effects of the ligature on mechanical sensitivity threshold. Inflammation was induced by a subplantar administration of 10 µl (½ diluted) of Complete Freund's Adjuvant (CFA, Sigma) under light anaesthesia. Secondary hyperalgesia induced by inflammation was verified by visual observation of paw oedema coupled with irregular gait. For back labelling of DRG neurons the proximal nerve stump was immersed several minutes in 0,9% saline solution containing 1% of Fluorogold (FG; Fluoro-Chrome Inc., Denver, CO, USA) and washed with 0.9% saline.

Intrathecal administration of neurotrophic factors or saline solution into the spinal subarachnoidal space at the S1 level of adult mice was done using a 30-gauge needle (BD Micro-fine). GDNF or NRTN (700 ng, AbCys) were injected once a day during 5 days starting one day before axotomy. Animals were sacrificed and lumbar DRGs and spinal cords were processed for immunohistochemistry and/or real time PCR. The efficiency of the injections on each animal was systematically monitored by analyzing IB4 staining (see below) in the dorsal horn of the spinal cord, which is normally lost after axotomy, but restored after injections of GDNF family ligands [16], [17].

### Cell culture

Neuron cultures were established from lumbar (L4–L5) dorsal root ganglia, three days after surgery, as previously described [18]. Briefly, ganglia were successively treated by two incubations with collagenase A (1 mg/ml, Roche Diagnostic, France) for 45 min each and then with trypsin-EDTA (0.25%, Sigma, St. Quentin Falavier, France) for 30 min. They were mechanically dissociated by passing several times through the tip of a fire-polished Pasteur pipette in Neurobasal (Life Technologies, Cergy Pontoise, France) medium supplemented with 10% fetal bovine serum and DNAse (50 U/ml, Sigma). Isolated cells were collected by centrifugation and suspended in Neurobasal medium supplemented with 2% B27 (Life Technologies), 2 mM glutamine and penicillin/streptomycin (20 U/ml, 0.2 µg/µl). Dissociated neurons were plated on d-polyornithine (0.5 mg/ml)–laminin (5 µg/ml)–coated glass coverslips and were incubated at 37°C in an incubator with a 95% air–5% CO_2_ atmosphere.

### *In Situ* Hybridization

PCR products of 400–600 bps derived from CaMK1a and ATF3 mRNAs were amplified from mouse DRG cDNA using specific primers, cloned into the pGEM-T easy plasmid vector using the TA cloning kit (Promega) and confirmed by sequencing. Antisense digoxigenin (DIG)-labeled murine riboprobes were generated using a DIG-RNA labelling kit (Roche Diagnostics), following the manufacturer's instructions and previously described protocol [4]. The primers used for generating the PCR products are described in Table 1. Adult injured ipsilateral, controlateral or naïve L4–L5 DRG, and brain were dissected in PBS and directly frozen and embedded in OCT compound (Tissue-Tek Miles, Elkhart, IN). Frozen slides were warmed to room temperature (RT) and then fixed in 4% paraformaldehyde (PFA) at 4°C 20 min followed by 5 min proteinase K and 15 min DEPC treated PBS treatments at RT. In situ hybridization was performed as previously described (Venteo et al., 2012). Slides were incubated with anti-DIG alkaline-phosphatase (AP)-conjugated antibody (Roche Diagnostics), washed, and revealed with NBT/BCIP staining. Negative controls were performed on sections with a sense probe.

**Table 1:** Primer sequences used for PCR products intended to generate In Situ Hybridization Probes (1) or for Real-Time Polymerase Chain Reaction (2).

Gene name	Orientation	Sequence (5′-3′)	Product length (bp)	Accession number
***CaMK1a*** (1)	ForwardReverse	AAGCACCCCAACATTGTAGC-AAGGCCTGCTCACAGGTAAA-	595	NM_133926
***CaMK1a*** (2)	ForwardReverse	TGGCTACCCACCCTTTTATG-CTTGATCTGCTCGCTCACTG-	247	NM_133926
**Atf3** (1)	ForwardReverse	CCACCCCACCTATCAAGGTA-GCTCAGAATGGACGGACAC-	425	NM_007498.3
**Atf3** (2)	ForwardReverse	ACAACAGACCCCTGGAGATG-CCTTCAGCTCAGCATTCACA-	187	NM_007498.3
**Sprr1a** (2)	ForwardReverse	CCAGCAGAAGACAAAGCAGA-GGGCAATGTTAAGAGGCTCA-	215	NM_009264.2
**Npy** (2)	ForwardReverse	TGGACTGACCCTCGCTCTAT-TGTCTCAGGGCTGGATCTCT-	187	NM_023456.2
**Polr2j** (2)	ForwardReverse	ACCACACTCTGGGGAACATC-CTCGCTGATGAGGTCTGTGA-	176	NM_011293.2
**Ddx48** (2)	ForwardReverse	GGAGTTAGCGGTGCAGATTC-AGCATCTTGATAGCCCGTGT-	205	NM_138669.1

### Immunohistochemistry

Frozen sections were prepared from adult DRG or sciatic nerve fixed in 2–4% PFA for 2 h at 4°C and cryopreserved 12–72 h in 25% sucrose at 4°C. DRG neurons in culture were fixed 15 min in 4% PFA and washed in PBS, 24 h after plating. The antibodies used were as follows: goat anti-Ret (R&D systems, (AF482) 1∶50), mouse anti-Neurofilament-200 (Sigma (N0142), 1∶1000), goat anti-GAP43 (Santa Cruz (sc-7458) 1∶500) rabbit anti-ATF3 (Santa Cruz (sc-188) 1∶500), mouse Beta 3 Tubulin clone tuJ 1 (MAB1195, R&D System, 1∶500). Rabbit anti-CaMK1 (Epitomics (2331-1), 1∶500) was used in this study. It must be noted that in mouse CaMK1 gene family nomenclature, CaMK1 is an alias of the CaMK1alpha isoform. Secondary antibody incubations were performed with Alexa Fluor-594 (Molecular Probes 1∶1000) or Alexa Fluor-488 (1∶500) conjugated secondary antibodies. Tissue sections were incubated with 10% donkey serum in PBS for 30 min at room temperature then incubated overnight at 4°C or 1 hour at room temperature with primary antibodies diluted in 0.3% donkey serum. Secondary antibodies were incubated for 1 hour at room temperature. For isolectin B4 (IB4) staining, cryosections were blocked in 1% BSA, 0.1% Triton in PBS for 1 h, and then incubated with IB4-Biotin (10 mg/ml, Sigma) and FITC-conjugated ExtrAvidin (Sigma, diluted 1∶400).Negative controls were run in routine by replacing specific primary antibody with normal serum of the same species as primary antibody. The slides were then washed in PBS before mounting with Mowiol medium. Images acquisition and analysis were done using a Zeiss LSM 5 confocal microscope.

### Cell counting

Immunolabelled neurons were counted on serial sections of 14 µm using ImageJ software. Areas and diameters of neuronal soma were measured using Metamorph software (version 7.1, Molecular devices). The number of neurons expressing the various molecular markers was determined by counting cells with neuronal morphology and clearly identifiable nuclei. As all counted neurons in our study were immuno-labeled in the cytoplasm or the nucleus, the identification of the nucleus was always possible. 7 slides (each containing around 10 sections) from DRGs were counted from at least 3 animals. The total number of positive neurons was determined. Given the fact that diameters of sensory neurons are comprised between around 10 to 50 µm and that on the same slide two serial sections are separated by a minimum of 100 µm it is impossible to double count a given neuron. When indicated on the graphs, the percentage of neurons expressing a given marker over the total number of a defined population was calculated.

### Real time PCR

Total RNA was extracted in TriReagent solution (Sigma) and treated with RQ1 DNase (Promega) according to manufacturer's instructions. Total RNA(1 µg) was reverse-transcribed with 100 U of Superscript II reverse transcriptase (Invitrogen) and 5 µM hexamer random primers (Boehringer Mannheim), 0,5 mM of each dNTPs (Pharmacia), 10 mM of dithiothreitol and 20 U of recombinant RNase inhibitor (Promega) 1 hour at 37°C and stored at −80°C until used. Real time PCR was carried out as described previously [4] using SYBR Green I dye detection on the LightCycler system (Roche Molecular Biochemichals). PCR reactions were carried out either in 384 well plates in a 5 µl volume containing 1.5 µl of RT product (final dilution 1/30), 0.5 µM of forward and reverse primers, and 1 µl of QuantiTect SYBR Green PCR Master Mix (Roche Diagnosis), or in capillaries (10 µl final volume). Amplified products were sequenced at least once (Millegene, France). The relative amounts of specifically amplified cDNAs were calculated using the delta-CT method [4], [19], [20] on three independent experimental replicates. The delta-CT method generates raw quantities that were subsequently normalised by dividing with an appropriate normalisation factor. This factor represents the geometric mean of the two most stable control genes (polymerase (RNA) II polypeptide J, Polr2j and DEAD box polypeptide 48, Ddx48), among eight tested in the different tissue samples investigated, and was calculated using genorm software (http://medgen.ugent.be/~jvdesomp/genorm/). Primer pairs used to generate PCR products are described in Table 1.

### *In vitro* quantification of neurite outgrowth speed

Neuronal cultures were established from lumbar (L4–L5) dorsal root ganglia of adult naïve or axotomized mice as described above, and seeded in four-well chambers at a density of 1000 neurons per well. We classified neurons as described by Smith and Skene (1997) [21]: neurons with processes with >1.5 branches/100 ìm were classified as arborizing, whereas those with <1.5 branches/100 ìm were classified as elongated. Neurons with neurites shorter than one cell diameter were classified as having no neurites. Time-lapse video microscopy was performed using an inverted Zeiss Axiovert 200 M equipped with a CCD camera (Micromax; Roper Scientific, Evry, France) and a motorized platine driven with MetaMorph 7.0 software (Molecular Devices, Downingtown, PA). At 2 h after plating, neurons were placed in the recording chamber and left to grow in the culture medium. Treatment of axotomised neurons with 0.5–1 µg/µl, 8-naphthoylene benzimidazole-3-carboxylic acid (STO-609, Sigma) was done for some experiments. Phase-contrast images of several neurons per well were collected with an LD A-Plan 20x/0.3 objective every 30 min for 24 h and analyzed off-line with the MetaMorph software. To measure the mean neurite growth speed of individual neurons, time lapse images were recorded every 30 min during 24 hours. Five to 10 neurites depending on the cell were analyzed. For each neurite the distance covered during a 3 hour period was calculated and the mean growth speed was expressed in micrometers per hour(µm/h).

### *In vivo* delivery of siRNA

Pools of three specific Small interfering RNAs (siRNA) (Silencer Predesign SiRNA, ID 186310 Ambion) against CaMK1a mRNA or non-targeting control siRNA (ON-TARGETplus Non-targeting Pool, Thermo Scientific) were used in this study and delivered by intrathecal injection as previously reported [22]. siRNAs (7 µg) were complexed with 1.8 µl of 200 µM linear low molecular weight PEI ExGen 500 (Euromedex, Souffelweyersheim, France). RNA-polymer complexes were allowed to form 10 min at room temperature. To allow visualization of transfected cells, 3 mM dextran-tetramethylrhodamine (Invitrogen, Cergy Pontoise, France) were added to the 5% glucose solution containing the RNA-polymer complex. 6–8 µl of the final solution (siRNA/PEI Exgen 500 *in vivo* transfection reagent complex) were injected at the S1 level of adult mice once a day for 5 days as described previously [22]. Animals were sacrificed, and lumbar DRGs were collected and processed for either immunohistochemistry or dissociated for time-lapse video microscopy.

### Statistical analysis

All data are expressed as the mean ± SEM. Depending on the nature of the experiment, statistical analyses were performed using, for two groups comparisons, Unpaired Student's *t* test or Mann–Whitney *U* test for small sample. For multiple group comparison one-way analysis of variance (ANOVA) and appropriate post-hoc tests Newman-Keuls or Dunnett were used. *p<0.05* was considered statistically significant.

## Results

### CaMK1a is transcriptionally induced in DRG after a sciatic nerve traumatism

**Figure 1:**
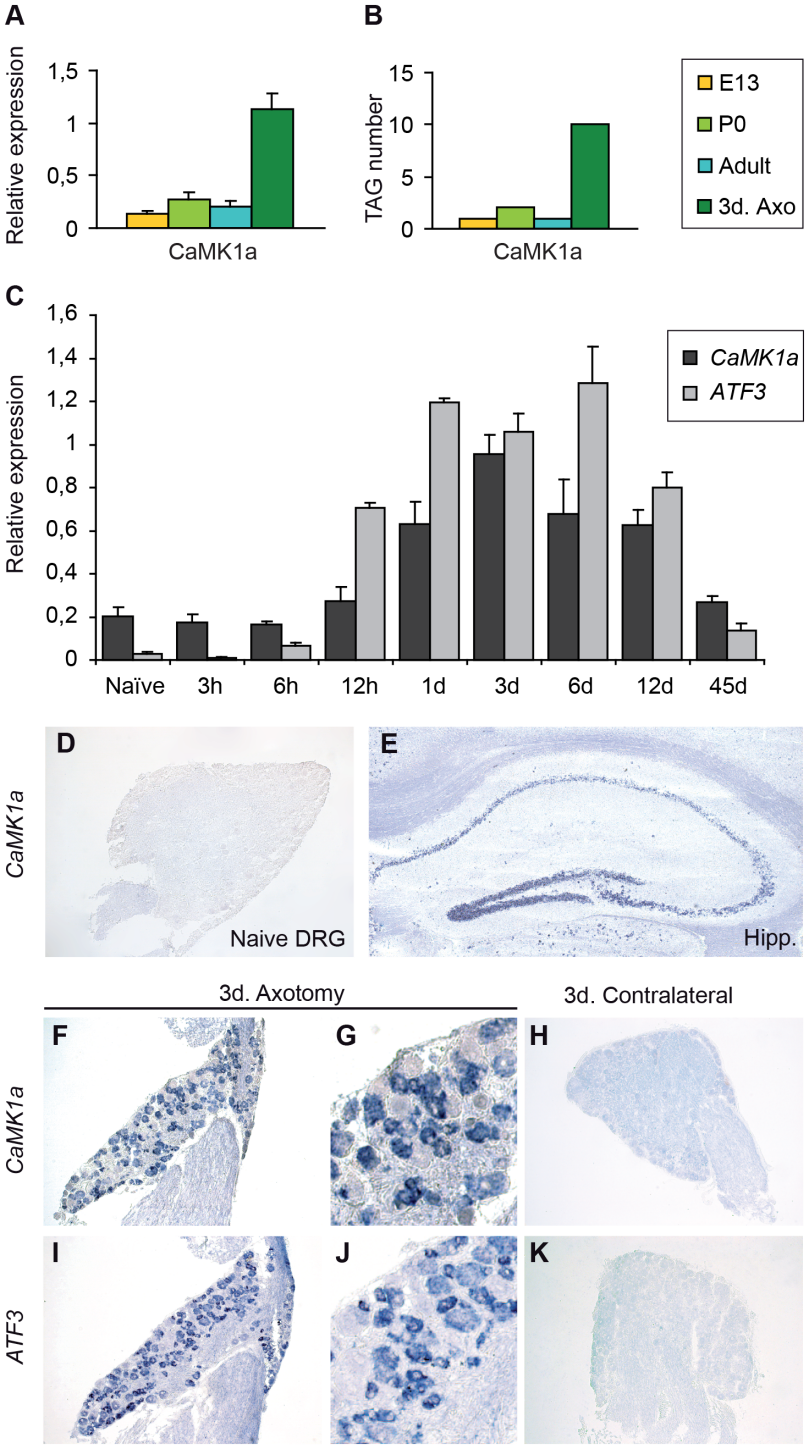
*CaMK1a* is induced in sensory neurons in mouse DRG after sciatic nerve axotomy. (**A**). Quantitative real-time PCR (QRT-PCR) analysis of the expression of *CaMK1a* mRNA at different stages of development (E13, P0, Adult) and three days (3d) after axotomy. A strong increase of the expression of *CaMK1a* after the nerve trauma compared to basal levels during development and in normal adult could be noted. (**B**). Results from SAGE (serial analysis of gene expression) analysis of mouse DRG at and three days (3d) after axotomy of the sciatic nerve. CaMK1a Tag numbers are low (1–2) at all stages of development and in normal adult, but increase to 10 Tags after axotomy. (**C**). QRT-PCR kinetics of expression of *CaMK1a* compared with that of *ATF3* shows that *CaMK1a* begins to increase at 12 h post-axotomy, peaks between 1–12 days and returns close to basal levels at 45 days. *ATF3* follows a similar pattern except that expression starts several hours before *CaMK1a*. (**D,E**). In situ hybridization on cryosections using a *CaMK1a* riboprobe showing the absence of expression in naïve adult DRG (**D**) and expression in the neuronal layers of the hippocampus (**E**). (**F–H**). In situ hybridization on DRG sections three days (3d) after axotomy of the sciatic nerve showing that *CaMK1a* mRNA is detected in neurons ipsilateral to the lesion (**F,G**) but is absent in contralateral DRG neurons (**H**). (**I–K**). As a positive control, mRNA detection by in situ hybridisation of the injury-induced gene *ATF3* shows that *ATF3* is induced only in neurons ipsilateral to the injury as expected (**I,J**) but not in contralateral neurons (**K**).

**Figure 2:**
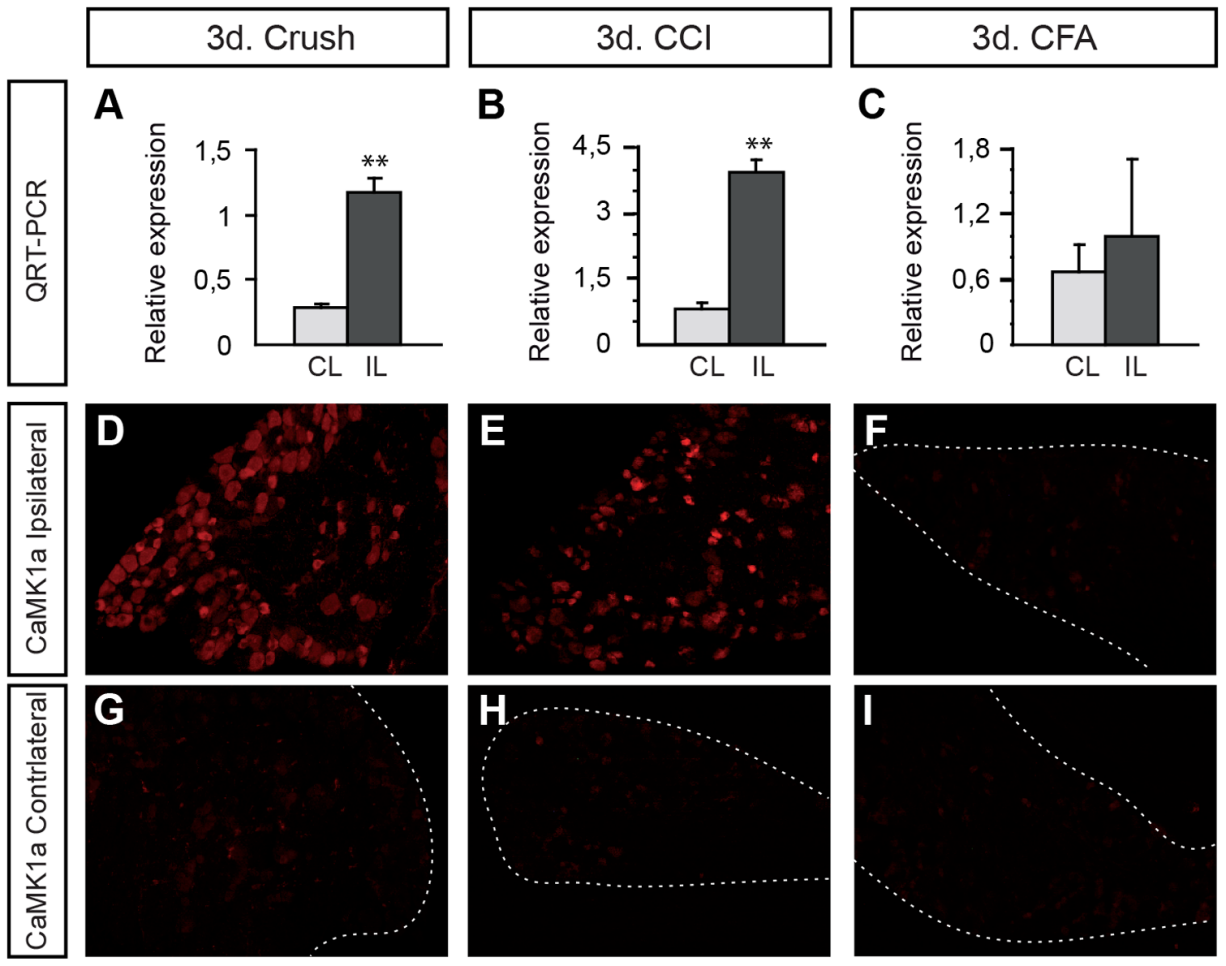
CaMK1a is induced in DRG neurons by nerve injury and not by inflammation. (**A–C**). QRT- PCR analysis of the expression of *CaMK1a* mRNA in DRG three days (3d) after crush, chronic constriction injury (CCI) or CFA induced inflammation, showing that *CaMK1a* mRNA is induced by crush and CCI but not by CFA injection. (**D–I**). Corresponding immunohistochemical staining for CaMK1a protein in the three experimental models showing sections of DRGs ipsi- (**D–F**) and contralateral (**G–I**) to the injury site. Note the presence of numerous strongly-labeled neurons in the ipsilateral DRGs from mice after crush (**D**) or CCI (**E**) but not CFA (**F**). Controlateral DRGs were CamK1a-negative for all conditions (**G–I**).

To follow the expression profile of *CaMK1a* in DRGs during development and after peripheral nerve injury, real time PCR analyses were carried out on mRNA extracted from DRG at E13, P0, adult (7–8 weeks) and three days after a sciatic nerve section in the adult (**Fig. 1A**). Results show that *CaMK1a* mRNA is strongly increased after a sciatic nerve axotomy compared to basal levels during development and in normal adult. (Note that this basal level of expression was not detectable at the protein level by immunohistochemistry **Fig. 2G-I** or ISH **Fig. 1D**). These quantitative RT-PCR results confirmed the data we previously obtained from SAGE (serial analysis of gene expression) analysis [4] (**Fig. 1B**). The other CaMK1 family members i.e. CaMK1beta, delta and gamma do not behave in the same way as CaMK1a and seem to be strongly expressed in the adult stage and poorly regulated after axotomy (data not shown). Analysis of the kinetics of *CaMK1a* induction showed that expression begins to increase at 12 h post-axotomy, peaks at 3 days and returns close to basal levels at 45 days. This expression profile paralleled that of the transcription factor ATF3, a marker of axotomized neurons commonly used as an indicator of DRG injury [23], except that the *ATF3* increase preceded that of *CaMK1a* by several hours (**Fig. 1C**).

To investigate the cell type expressing *CaMK1a*, we carried out in situ hybridization using DIG-labelled probes. We confirmed that no signal was found in naïve DRG (**Fig. 1D**). As a positive control, *CaMK1a* mRNA was detected in adult brain sections (**Fig. 1E**) as described previously [12]. On adult mouse L4–L5 DRG sections three days post-axotomy, *CaMK1a* expression is apparent in a sub-population of neurons ipsilateral to the nerve axotomy (**Fig. 1F,G**). No signal was found in DRGs contralateral to the injured nerve (**Fig. 1H**). Similarly and as expected, *ATF3* is up regulated in ipsilateral DRG after axotomy (**Fig. 1I,J**) but not in the non-axotomized controlateral DRG (**Fig. 1K**).

Altogether these data demonstrate the de novo induction of the *CaMK1a* gene in a subpopulation of DRG neurons after a nerve traumatism.

### CaMK1a is specifically induced by axonal damage and not by a perineural inflammation

To see if *CaMK1a* is induced in other models of peripheral nerve injury, we analyzed CaMK1a expression after crush, spinal nerve ligation (CCI) and peripheral injection of Complete Freund's adjuvant (CFA). A similar *de novo* induction of *CaMK1a* mRNA was observed by real time PCR three days (3d) after a crush lesion (**Fig. 2A**). Loose ligation of the sciatic nerve, a model of chronic nerve compression also caused up-regulation of *CaMK1a* mRNA in the DRG (**Fig. 2B**). However, CFA injection into the hindpaw caused no increase of *CaMK1a* (**Fig. 2C**). These QRT-PCR results were confirmed and extended by immunohistochemistry on DRG sections using an antibody directed against CaMK1a. Strongly-labeled CaMK1a-positive neurons were observed in the DRGs from crush and CCI injury models (**Fig. 2D,E**). In the CFA treated animals, a few weakly stained neurons could be observed (**Fig. 2F**). Little or no staining was visible in the contralateral DRGs for all conditions (**Fig**. **2G,H,I**). These results show that *CaMK1a* expression is specifically induced by mechanical nerve injury but not by inflammation.

### Preferential induction of *CaMK1a* in Ret+ sensory neurons after peripheral trauma

**Figure 3:**
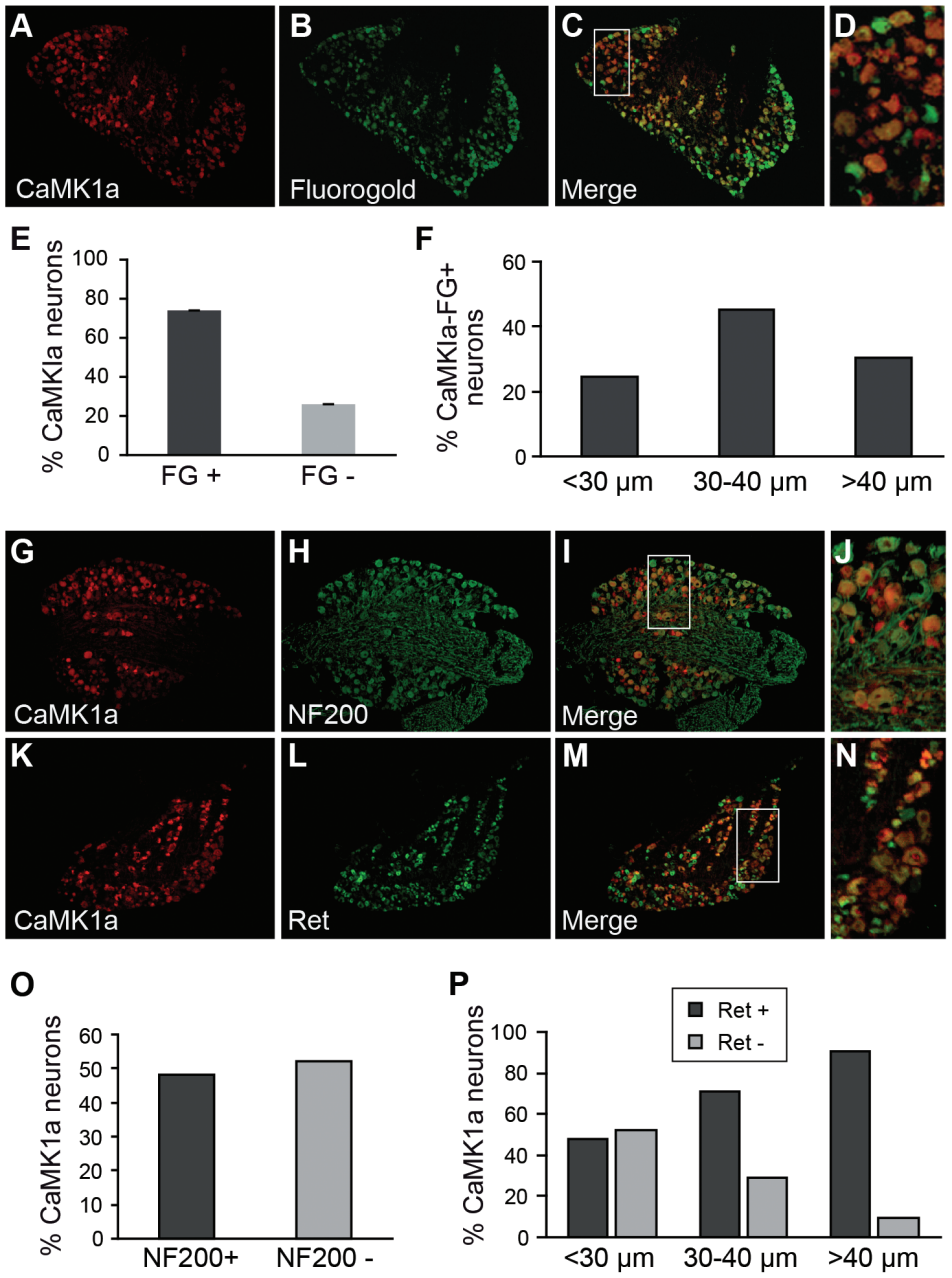
CaMK1a is preferentially induced in large diameter Ret+ neurons after axotomy. (**A–D**). Combined CaMK1a immunohistochemistry and retrograde labelling with Fluorogold (FG) on L4–L5 DRG sections three days post-axotomy of the sciatic nerve. FG was applied at the cut nerve stump and specifically labels axotomized neurons. (**E**). Counts on DRG sections show that 74+/−2% of CaMK1a+ neurons are FG+. (**F**). Cell soma size distribution of CaMK1a+ neurons in DRG after sciatic nerve axotomy. (**G–J**). Double-immunofluorescent staining for CaMK1a and NF-200 on sections of L4–L5 DRG three days post-axotomy. (**K–N**). Double-immunofluorescent staining for CaMK1a and Ret on DRG sections three days post-axotomy shows numerous co-labelled neurons for both proteins. (**O**). Counts of CamK1a+NF200+ double-labeled cell reveals that about 50% of CaMK1a-positive neurons are NF200+. (**P**). Size repartition of CamK1a+Ret+ double-labeled cells showing that the vast majority of CaMK1a-positive neurons with medium-large cell soma diameter also express Ret.

To gain more insight into the population of neurons concerned by the up-regulation of *CaMK1a* expression, we first carried out retrograde labeling of DRG neurons using the tracer Fluorogold (FG) applied at the site of nerve transection combined with CaMK1a immunohistochemistry. Counting of CaMK1a immunopositive neurons (**Fig. 3A**), FG-positive (**Fig. 3B**) and double-labeled neurons (**Fig. 3C–D**) showed that close to 75% of CaMK1a+ neurons were FG-positive (**Fig. 3E**) confirming the strong association of its expression with the traumatic state.

Next, cell size distributions and co-localization studies with known markers for neuronal subtypes were undertaken (**Fig 3G–P**). CamK1a+ neurons were distributed across all size classes, with the majority being of medium (30–40 µm) cell diameter (**Fig 3F**). NF200+ neurons are myelinated afferents and comprise 30% of the neurons in the L4/L5 DRG [24], [25]. Double labeling with NF200 showed that 47% of CaMK1a+/FG+ neurons were NF200+ (**Fig 3G–J,O**). About 60% of DRG neurons are Ret+ and the majority of these neurons are non-peptidergic nociceptors [16]. However, a sub-population of Ret+ neurons has med-large diameter cell soma and belongs to specific sub-types of low-threshold mechanoreceptors afferents [26]–[28]. Double-labeling with Ret showed that about 48% of small soma (<30 µm), 71% of 30–40 µm diameter and 91% of >40 µm diameter Ret+ neurons were CaMK1a-positive (**Fig 3K–N,P**). Overall, these results demonstrate that CaMK1a is preferentially albeit not exclusively induced in axotomized neurons that also express Ret.

### CaMK1a is localized to soma and neurites of sensory neurons in culture and along the fibres of regenerating Ret+ neurons *in vivo*

**Figure 4:**
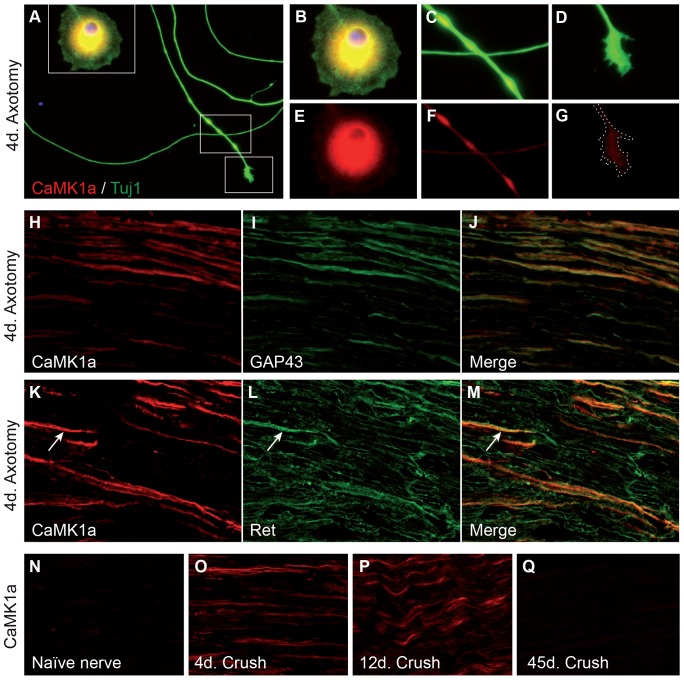
CaMK1a is localized to soma and neurites of axotomized DRG neurons. (**A–G**). Double-immunofluorescence for CaMK1a (A,E,F,G) and the neuronal marker Beta 3 tubulin (Tuj1) (A,H,I,J) on 24 h cultures of neurons from L4/5 DRG ipsilateral to sciatic nerve axotomy carried out 3 days before (thus referred to four days (4d) Axotomy). Strong CamK1a expression was detected in the cytoplasm of axotomized DRG neuron (**inset in A, B,E**) and along their neurites (**A**,**C,F**). A weaker expression was also observed in the central part of a DRG growth cone (**A,D,G**). (**H–M**). CaMK1a immunoflurorescent labeling of nerve fibres on longitudinal sections of the proximal part of the sciatic nerve four days (4d) after axotomy, combined with GAP43 (H–J) or Ret (K-M) showing numerous double-labeled fibers using both markers. (**N–P**). CaMK1a labelling on longitudinal sections of a naïve sciatic nerve (**N**) and of sciatic nerves just distal to a crush injury at 4 (**O**), 12 (**P**), and 45 (**Q**) days post-lesion, showing no labeling in naïve nerve, strong labeling in fibres at 4- and 12 days and a marked reduction after 45 days.

To study the subcellular localisation of CaMK1a, immunohistochemical studies were performed on sensory neurons placed in culture 3 days after sciatic nerve section, and allowed to grow for 1 more day. Axotomized neurons were identified by their characteristic elongated growth clearly visible in culture [21]. Neurons, identified by Tuj1 (Beta 3 tubulin) labeling (**Fig. 4** A–G), exhibited a strong CaMK1a labelling in soma (**Fig. 4E**) and neurites (**Fig. 4F**). A light labelling was also observed in the growth cone centre but did not extend to filopodia structures (**Fig. 4G**).

To see if CaMK1a was localized to growing fibres of regenerating neurons *in vivo*, we carried out double labeling for CaMK1a and GAP43, a marker of regenerating neurites [29], on longitudinal sections of sciatic nerve that had undergone a crush injury 4 days earlier. Co-localisation with GAP43 showed that CaMK1a protein is present along regenerating fibres after trauma (**Fig. 4H–J**). In line with the strong co-localisation of CaMK1a and Ret in DRG neuron cell bodies, numerous double CaMK1a/Ret positive fibres were also observed in injured sciatic nerve (**Fig. 4** K–M). Ret+ fibres were either abundant, thin and lightly-stained fibres or sparse large-diameter heavily-stained fibres. CaMK1a staining was generally associated with the large strongly stained Ret+ fibres. Previous studies showed that *Ret* expression in DRG sensory neurons is not dramatically altered by sciatic nerve injury and *Ret* mRNA is absent in both normal and axotomized sciatic nerve [30]. No CaMK1a labelling was observed in naïve mouse sciatic nerve (**Fig 4**. N). Labeling for CaMK1a protein in the proximal region of the sciatic nerve at 4, 12 and 45 days after crush (**Fig. 4** O–Q) showed strong labeling of fibres at days 4 and 12, with a markedly reduced staining at day 45 post-lesion, a pattern of expression similar to that of *CaMK1a* mRNA in the DRG (see **Fig. 1C**). Altogether, the *in vitro* and *in vivo* results suggest that CaMK1a is present along regenerating fibres of injured DRG neurons and appears to be preferentially associated with Ret+ fibres.

### GDNF family members influence the *de novo* expression of *CaMK1a* in DRG neurons

We next examined if the interruption of target-derived neurotrophic signals might drive the up-regulation of *CaMK1a* expression. As stated above, most of the CaMK1a positive neurons express Ret which constitutes the receptor signaling component for the neurotrophic factors of the glial-derived neurotrophic factor (GDNF) family, including GDNF [31] and Neurturin (NRTN) [32].

**Figure 5:**
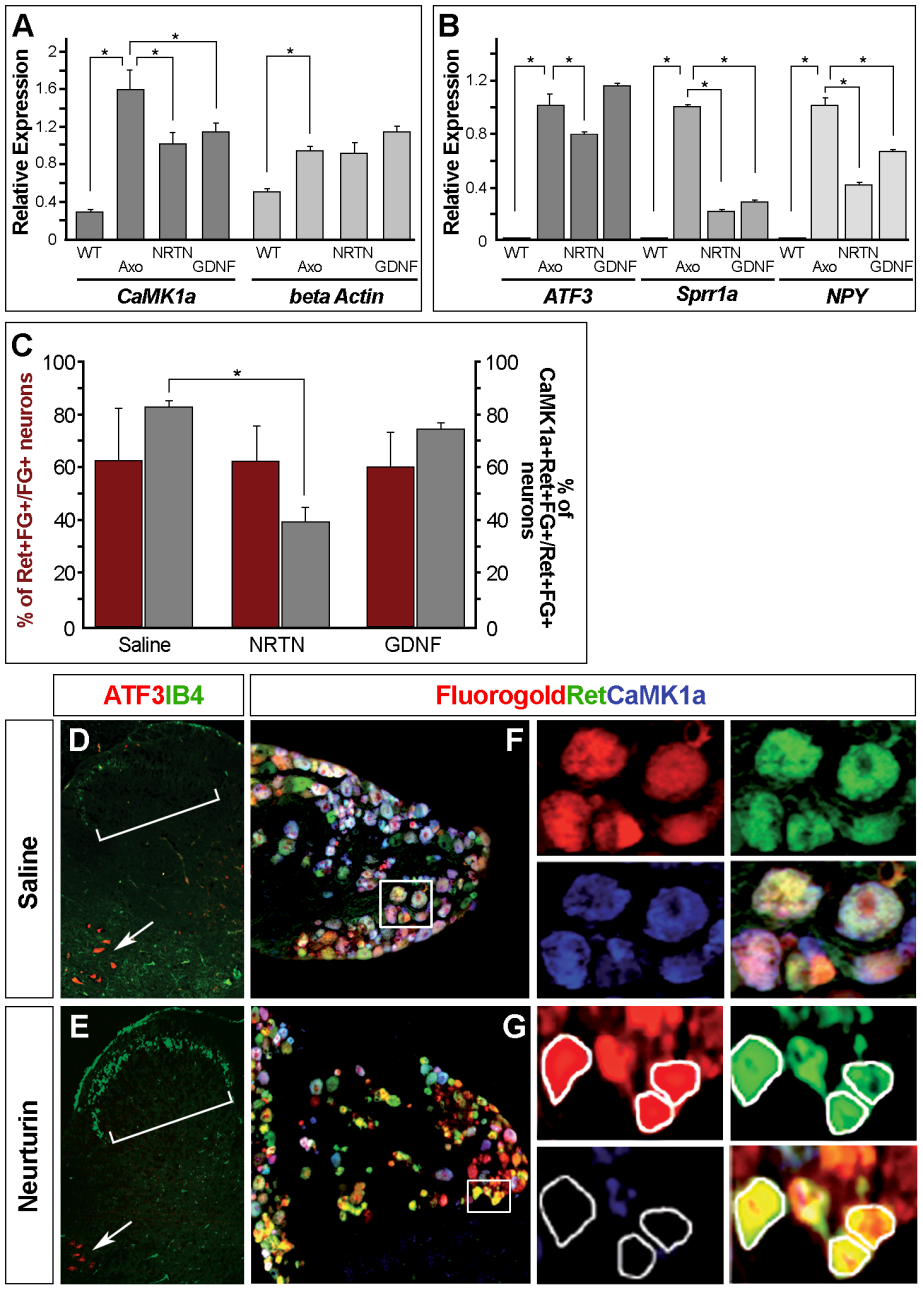
GDNF and NRTN delivery partially normalizes the *de novo* expression of *CaMK1a* in DRG neurons. (**A–B**). QRT-PCR analysis of the expression of *CaMK1a* and *Beta Actin* (**A**) or *ATF3*, *Sprr1a* and *NPY* (**B**) mRNA in naive (**WT**) or axotomized (**Axo**) DRGs compared to axotomized DRG after GDNF or NRTN intrathecal delivery. Statistically relevant differences (p<0.05) are signified with an “*”in the graphs. (**C**). Red columns on the graph (left scale) show that the percentage of Fluorogold(FG)+Ret+ neurons over the total number of FG+ neurons remains stable - around 60% - in axotomized DRG after intrathecal injections of either a saline solution, Neurturin (NRTN) or GDNF, thus establishing the Ret+FG+ neurons as a reliable reference population. Dark grey columns on the graph (right scale) show the percentage of CaMK1a+ FG+Ret+ neurons over the total number of FG+Ret+ neurons in the three conditions described above. In saline treated DRGs close to 81% of all Ret+/FG+ neurons are also CaMK1a+. This percentage significantly drops to 38% after NRTN delivery. In GDNF-treated DRGs there a is a slight reduction to 74% which is however below the threshold of significance. (**D,E**). Double labeling of ATF3 and IB4 on adult spinal cord transverse sections from mice having undergone a sciatic nerve axotomy and injections of either a saline solution (D) or NRTN (E). Only the ipsilateral axotomized side, revealed by the motoneuronal expression of ATF3 (arrows) is shown. The IB4 staining in the dorsal horn of the spinal cord is greatly reduced in animals injected with a saline solution (bracket in D) while it remains strong in animals injected with NRTN (bracket in E). (**F,G**). Illustration of a double-immunofluroscence experiment used for the quantification, using CaMK1a and Ret antibodies combined with FG detection on transverse sections of axotomized DRGs from animals injected either with a saline solution (F) or NRTN (G). On the right panels are shown close-ups corresponding to the white frames in the enlarged images, showing individual labeling (Fluorogold in red, Ret in green and CaMK1a in blue) and the merged image. Note the presence of many Ret+FG+CaMK1a+ yellow cells in the NRTN injected animals which are rare in the controls.

Injections of NRTN or GDNF significantly inhibited the marked increase of *CaMK1a* mRNA induced by the lesion, with NRTN being a little more potent (**Fig 5A**). As expected beta-actin mRNA was up regulated after axotomy [6], however its expression level was not affected by GDNF family members (**Fig 5A**). Quantification studies were also performed on several other known de novo trauma-induced transcripts such as ATF3, the small proline-rich protein Sprr1a or the neuropeptide Y (NPY) [4], [6] (**Fig 5B**). Both NRTN and GDNF partially corrected the expression levels of *Sprr1a* and *NPY*, with NRTN appearing more potent than GDNF. NRTN was also able to significantly reduce *ATF3* expression. In contrast, in our experimental paradigm, GDNF was not able to do so, which appears in contradiction with data reported by Averill et al (2004) [33] (see discussion).

These data show that injections of GDNF family members reduce the de novo induction of *CaMK1a* mRNA in parallel to several other known transcripts after a sciatic nerve trauma. These reduced expressions remain however significantly higher than the basal expression levels of these mRNA.

The QRT-PCR results were, at least in part, supported by co-labeling immunohistochemical analyses. Fluorogold (FG) was applied to axotomized sciatic nerve in order to back label axotomized neurons and solutions containing either NRTN, GDNF, or saline were delivered to the subarachnoid space by intrathecal injection. To check the efficiency of the intrathecal injections on each animal, IB4 staining in the dorsal horn of the spinal cord, which is normally lost after axotomy, but restored after injections of GDNF family ligands, was systematically analyzed (**Fig 5D,E**).

In these experiments - to avoid potential bias due to variability during the surgical manipulation, and since we assessed putative effects of Ret ligands on CaMK1a expression -, we used Ret+FG+ neurons as the reference population. Indeed, we found that the percentage of Ret+FG+ neurons over the total number of FG+ cells remains remarkably stable in all three conditions (63+/−3,75; 60+/−2,5 and 63+/−2,2 for saline GDNF and NTRN respectively; **Fig. 5D**, left axis). This further confirmed that Ret expression is largely unaffected after axotomy and by administration of its ligands thus establishing this population as a reliable reference [30]. In DRG from mice injected with saline solution, close to 81%+/−2,56% of all Ret+FG+ neurons were also CaMK1a+ (**Fig. 5C and D**). This percentage significantly dropped to 38%+/−6,31 when mice were treated with NRTN (**Fig. 5C and E**), visualized on DRG sections by the presence of numerous “Ret+FG+CaMK1a” yellow cells in **Fig 5G** which are rare in the control condition (**Fig. 5F**). In mice injected with GDNF, a slight reduction of this percentage was also observed compared to control animals (74%+/−3,02) which remains however under the threshold of significance. Thus despite a clear effect at the mRNA level administration of GDNF appears less efficient than NRTN in reducing the number of detectable CaMK1a-immunolabelled neurons after 3 days post-axotomy.

Altogether, our results support the view that the loss of retrograde signalling from peripheral targets is one of the mechanisms by which CaMK1a is *de novo* induced in injured sensory neurons, and that its expression is negatively regulated by the Ret signalling pathway notably triggered by NRTN.

### Interference with the CaMKK-CaMK1a pathway reduces the speed of neurite growth of injured sensory neurons

Considering the expression pattern of *CaMK1a* in DRG neurons after a peripheral nerve trauma, and the fact that this molecule influences axonal growth in cortical neurons (reviewed in [14]), we investigated its potential role in the intrinsic capacities of sensory neurons to regrow after a lesion. The speed of neurite outgrowth was investigated in L4–L5 DRG neurons dissected and placed in culture.

In preliminary experiments [18], [22], the Fluorogold retrotracer (FG) was applied to the cut stump of transected sciatic nerve and corresponding L4/L5 DRG neurons were put in culture three days later, in parallel to “naïve” sensory neurons from control animals. In naïve cultures 100% of sensory neurons had either no neurite or display an arborized growth mode with extensive branching and modest neurite length 24 hours post plating. In cultures from axotomized animals, 60% of DRG neurons had long, sparsely branched axons typical of the “regenerating” mode of growth stimulated by prior nerve injury and described by Smith and Skene 1997 [21]. We then classified neurons as previously described [18], [21]. Neurons with processes with >1.5 branches/100 ìm were classified as arborizing, whereas those with <1.5 branches/100 ìm were classified as elongated. Neurons with neurites shorter than one cell diameter were classified as having no neurite. Moreover we showed that all elongated growth neurons were FG labeled i.e pre-axotomized.

**Figure 6:**
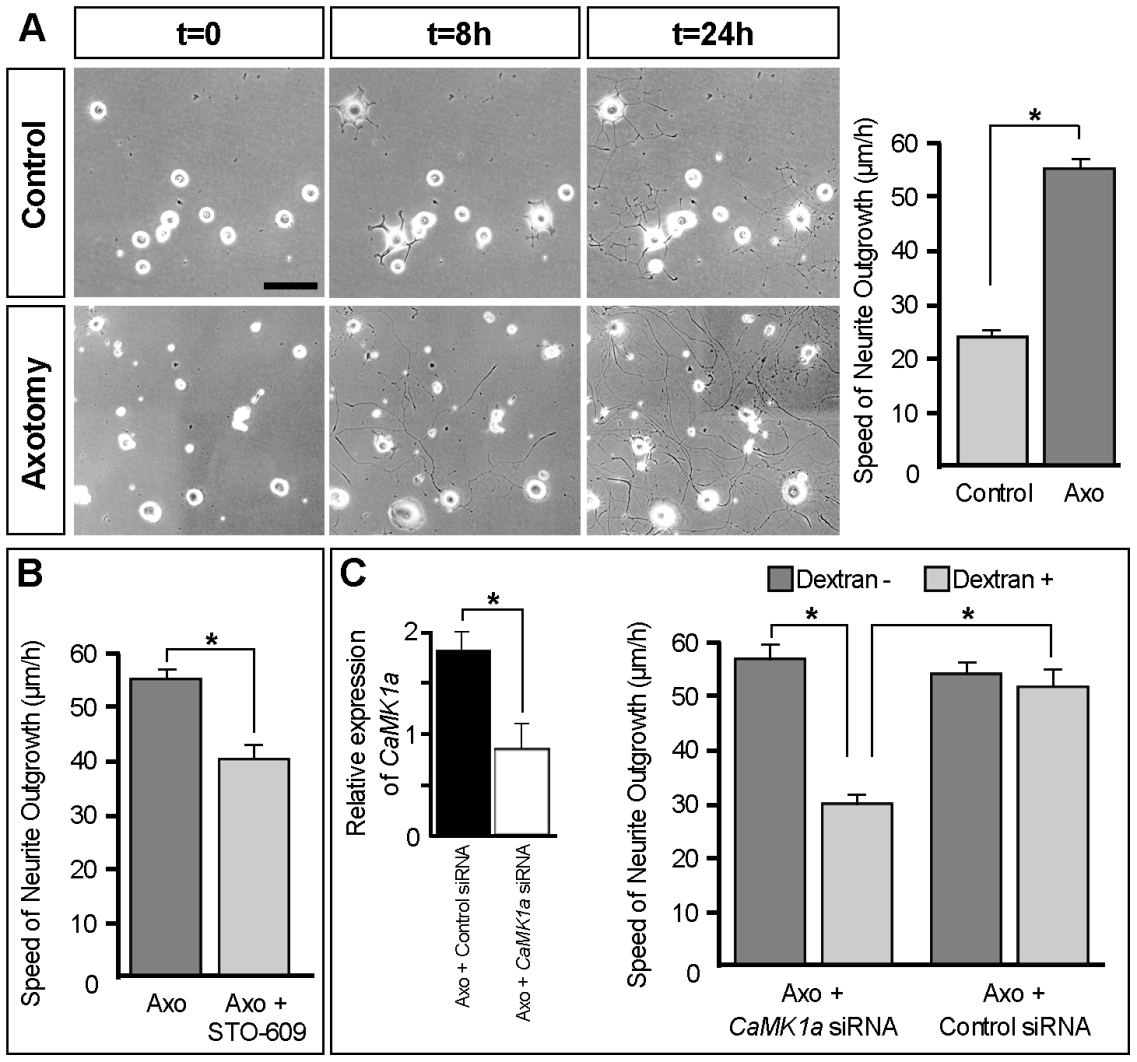
Perturbation of the CaMKK-CaMK1a pathway reduces neurite growth velocity of injured DRG neurons *in vitro*. (**A**). Phase-contrast illustrative images at 0, 8 and 24 hours after plating of sensory neurons dissected from naïve animals (upper panels) or from mice having undergone a sciatic nerve axotomy 3 days before (lower panels). Scale bar  = 100 µm. Note that after 24 h, sensory neurons from controls exhibit an arborized growth while many axotomized neurons exhibit an elongated growth. The graph on the right illustrates the growth speed of neurons in both conditions. In controls, the arborized neurons extend neurites at a velocity of 24.92 µm/h+/−1.96, while axotomized elongated neurons extend neurites at a velocity of 54.7+/−2.2 µm/h, confirming previous published studies. (**B**). Quantification of the neurite growth velocity of axotomized elongated neurons put in culture 3 days after a sciatic nerve section during 24 hours without (dark grey column) or with (light grey column) treatment with the CaMKK inhibitor STO-609. Untreated axotomized elongated neurons normally extend neurites at a velocity of 54.7+/−2.2 µm/h. With STO-609 (0.5 µg/µl) treatment, we observed a 25% reduction of the growth speed which drops to 40.8+/−2,6 µm/h. (**C**). Quantification of the effect of Control or CaMK1a siRNA on the velocity of neurite outgrowth of axotomized elongated neurons. Mice were given intrathecal injections of CaMK1a siRNA or control non-targeting siRNA in transfection agent containing dextran- tetramethylrhodamine as an indicator of transfection. The graph on the left show QRT-PCRs revealing a 46% reduction of CamK1a expression specifically in neurons injected with CamK1a siRNA compared to control siRNA. The neurite growth velocities of axotomized dextran+ and dextran- neurons were evaluated and reported on the graph on the right. CaMK1a siRNA transfection reduced DRG neurite outgrowth from 55+/−2,48 to 30+/−2,47 µm/h while control siRNA had no effect.

We first confirmed previous data showing that neurons displaying an elongated growth profile exhibit a growth velocity significantly higher than arborized neurons (54.7+/−2.2 *versus* 24.92 µm/h+−1.96) [22] (**Fig. 6A**). The morphological characteristic of elongated growth illustrated in **Fig. 6A** was used for off-line analysis neurite growth of axotomized neuron in subsequent experiments.

To assess a putative role of the CaMK1a protein in influencing the regenerative growth capacities of axotomized elongated sensory neurons, we first used an *in vitro* cell-permeable and selective inhibitor of the CaMKKs called 1,8-naphthoylene benzimidazole-3-carboxylic acid (STO-609) [34], [35]. Indeed previous studies have demonstrated that CaMK1 and CaMK4 belong to the CaM kinase cascade and are activated by phosphorylation of an activation loop Thr residue by the upstream CaM kinase kinase (CaMKK) [36], [37].

The growth characteristics of naïve DRG neurons in culture were not affected by STO-609 (data not shown). In contrast, a substantial decrease (from 54.7+/−2.2 µm/h (n = 14) to 40.8+/−2.6 µm/h (n = 18); 25% reduction) in the neurite outgrowth speed could be noted on axotomized DRG neurons displaying an elongated growth profile treated with STO-609 during the first 24 h (**Fig. 6B**).

Next, to see if specific inhibition of *CaMK1a* affected neurite growth in axotomized elongated neurons, a pool of CaMK1a siRNAs complexed with transfection reagent and loaded with dextran tetramethylrhodamine was injected intrathecally just before, during and after sciatic nerve axotomy. In previous experiments [22] using this protocol, we evaluated the siRNA transfection efficiency to be 50–80%. *CaMK1a* mRNA levels were compared in mice injected with control siRNA and CaMK1a siRNA. CaMK1a siRNA injection resulted in 46 ± 5% (*n* = 3) decrease of *CaMK1a* mRNA in lumbar axotomised DRG (**Fig. 6C, left panel**), demonstrating the efficiency of the CaMK1a siRNA pool used in this experiment. SiRNA transfected axotomized neurons were identified by their neurite growth profile combined with dextran-tetramethylrhodamine labeling. Uptake of dextran was used as an indicator of successful transfection. [22]. Dextran-positive axotomized elongated neurons transfected with *CaMK1a* siRNA exhibited a decreased outgrowth speed compared to dextran-negative axotomized elongated neurons (from 55+/−2.48 µm/h in dextran negative neurons (n = 12) to 30+/−2.47 µm/h in dextran positive neurons (n = 12); 45% reduction) (**Fig. 6C, right panel**). Note that the observed decrease in growth velocity is probably an underestimate, since for technical reasons our analysis was restricted to neurons that displayed observable elongated growth. If CaMK1a siRNA completely blocked neurite outgrowth in some neurons, they would not be included in the analysis. As a negative control, the same procedure was carried out with a control siRNA having no known target. No difference in neurite growth velocity was observed between dextran-positive (n = 14) and dextran-negative axotomized elongated neurons (n = 16) in this case (**Fig. 6C**). Altogether, these data indicate a role for the CaMKK-CaMK1a pathway in the intrinsic growth capacities of axotomized DRG sensory neurons exhibiting an elongated growth profile *in vitro*.

## Discussion

Injury to peripheral nerve causes a dynamic and complex pattern of transcriptional and post-transcriptional changes in somatosensory neurons of the DRG. These changes result from a combination of the direct effects of the mechanical insult, loss of peripheral signals, as well as novel signals at the injury site triggered by molecules synthesized by glial and immune cells during Wallerian degeneration distal to the nerve injury ([2], [38] for review). An important challenge is to understand the precise roles of the various molecular actors in the neuronal response i.e neuroprotection, regeneration and target re-innervation, but also in the development of neuropathic pain that is often associated with peripheral nerve damage.

Here we show that the calcium calmodulin kinase CaMK1a represents a new injury-induced marker of mechanical peripheral nerve injury in a subset of neurons. Its post-traumatic induction results at least in part from the interruption of target-derived retrograde signals. Functionally, the CaMKK-CaMK1 intracellular signalling pathway appears to play a role in the intrinsic growth capacity of peripheral neurons following injury. Thus, this signalling pathway represents a new actor in the peripheral nerve injury response.

Expression kinetic studies revealed that *CaMK1a* transcripts are *de novo* induced in DRG neuron after nerve injury. Expression increases several hours after a sciatic nerve section and remain high until 12 days after the damage, suggesting a role in the medium-term post–traumatic regenerative events. Moreover its expression was found to be associated with the lesion state as attested by the use of Fluorogold retrotracer. Peripheral nerve transection is the most severe traumatism; it induces a loss of nerve continuity and as a consequence, a neuroma forms at the distal end of the proximal stump comprised of injured axons along with proliferating Schwann cells, fibroblasts and sympathetic axons [1]. However, the induction of *CaMK1a* expression is not dependent upon the type or the severity of nerve trauma. We investigated its expression in other models of nerve injury i.e acute or chronic compression in which epi- and perineurium tubes are usually preserved and promote regenerative fiber growth. These damages are less severe models of trauma [39], but they lead to a similar high induction of *CaMK1a*. By contrast, a severe inflammation induced by CFA injection into the hindpaw which causes oedema, infiltration of neutrophils and synthesis and release of algogenic and sensitizing molecules [40], [41] did not induce *CaMK1a*.

We thus conclude that even though nerve damage is also accompanied by immune and inflammation components at the lesion site [42], these processes appear not to be key inducers of *CaMK1a* expression. A critical point in this *de novo* induction seems to be the mechanical insult produced by the trauma. However, it must be noted that our retro labelling experiments showed that a small proportion of CaMK1a positive neurons were not Fluorogold labelled. An inefficient retro labeling of those neurons or an induction of CaMK1a by regulatory factors released by degenerative fibers cannot be excluded. However we could also hypothesize that the fibers of those neurons do not project into the sciatic nerve and thus were not exposed to FG after axotomy but were sectioned by the surgical procedure. Such a mechanical-dependant induction has already been described for ATF3, another injury induced gene known to contribute to the intrinsic growth state of neurons [8]. Indeed several studies show that the induction of *ATF3* after injection of different noxious chemical stimuli is related to the potential of these stimuli to induce nerve damage [43], [44] as we suggest to be the case for CaMK1a. Another argument in favour of this hypothesis results from our *in vitro* studies. Neurons in culture are thought to behave as *in vivo* axotomised neurons as they rapidly express classical Regeneration Associated Genes [45]. While *CaMK1a* was never detected at the protein level in DRG at any stages of development it was found to be highly and rapidly induced in DRG neurons in culture from E13, neonatal or adult naïve DRG (data not shown). These results reinforce our postulate that *CaMK1a* represents a new specific marker of sensory neurons lesion.

Initiation of changes in gene transcription in injured DRG neurons have been proposed to be the consequences of damage signals acting through distinct mechanisms, including release of factors at the lesion site and interruption of retrograde signals [2], [38].

In the present study we have uncovered the rapid induction of CaMK1a preferentially in the Ret+ injured DRG neurons. We thus investigated the effects of exogenous infusion of Ret ligands GDNF and NRTN on injury induced *CaMK1a* expression by intrathecal injection after a mechanical nerve lesion and showed that both are able to counteract this process at the mRNA level. The effect of GDNF which preferentially binds the Ret-GFRα1 receptor complex is consistent with the fact that *Gfra1* is increased after nerve injury [17]. The effect of exogenous NTRN on *CaMK1a* expression might appear more surprising since several studies have shown that the expression of its preferred co-receptor, *Gfrα2*, is down-regulated by axotomy [17], [46]. However, although NRTN has the highest affinity for GFRα2, it binds GFRα1 to a lesser extent [47]. In addition, recent data demonstrate that the biology of GDNF family ligand (GFL) signaling is much more complex than originally assumed. Indeed, GFLs can signal in neuronal and glial cells independently of Ret in collaboration with other transmembrane proteins like NCam or B1 integrin [48], [49]. Moreover, investigations from Schmutzler et al. (2011) [49] on the role of GFLs in neuronal sensitization on sensory neurons in culture, suggest that the NCam-dependent actions of NRTN may be mediated by the direct binding of NRTN to NCam, [46]. Intriguingly, in our experimental paradigm, NRTN appears more potent than GDNF in counteracting the de novo induction of CaMK1at the messenger level but also and particularly at the protein level. This is also the case for several other lesion-induced markers, including *Sprr1a* or *NPY*. The reasons for such observations remain unclear but might reflect the complex modes of action of the GFLs after trauma. In addition, in our hands, NRTN injections, but not GDNF, were able to significantly reduce the expression of *ATF3* after nerve injury. This result appears contradictory to previous studies performed on rats [33]. However, it must be noted that we performed these experiment on mice and that our protocol differs considerably form that of Averill et al in its duration (5 days *versus* 14 days) and mode of delivery (multiple injections *versus* pump delivery) which might explain this discrepancy.

Nevertheless, whatever the mode of action of the GFLs, our data support the hypothesis that the loss of retrograde neurotrophic transport from peripheral targets to the DRG neuron cell body is one of the signals that induce *CaMK1a* expression.

The role of neurotrophic factors in phenotypic changes occurring after a trauma has been greatly investigated. Indeed, several studies have shown that exogenous Ret ligand GDNF can prevent neurochemical changes in injured sensory neurons and has an antiallodynic effect on neuropathic pain behaviours induced by peripheral nerve injury [33], [50]–[52]. In addition, we have recently shown that intrathecal injection of GDNF or NTRN arrested the axotomy-induced down-regulation of the NaKATPase modulator, FXYD2, in *Ret* expressing nociceptors [53]. Furthermore application of NGF to injured nerves suppresses the induction of injury-induced molecules [54], [55] and peripheral application of NGF antibodies causes the appearance of axotomy-like changes in intact sensory neurons [56]. Thus it appears that access to neurotrophic factors is essential for the maintenance of normal adult phenotype.

Our study provides some hints about the rationale of CaMK1a induction in injured DRG neurons. Indeed, *in vivo* administration by intrathecal injections of a pool of small interfering RNA (siRNA) specifically directed against *CaMK1a* mRNA, followed by plating of DRG neurons, leads to a significant reduction of the growth speed of injured neurons which exhibit an elongated growth profile in culture. This is also consistent with our observation that STO-609, a selective inhibitor of the CaMKKs [34], has a similar effect in an *in vitro* regenerative growth assay of sensory neurons. It is of note that, to avoid any bias notably inherent to the culture procedures during which some neurons die or fail to extend processes and since our primary aim was to address a putative role for CaMK1a in the regenerative growth capacities of sensory neurons, we have chosen to focus our functional analysis specifically on neurons exhibiting an elongated growth profile. By doing so, we found that interfering with CaMK1a in other processes initiated in injured neurons. However, in our experimental paradigm we cannot exclude putative roles for CaMK1a in other processes iniated in injured neurons. Nevertheless, our data support a role for CaMK1a in the regenerative growth of sensory neurons after a mechanical nerve lesion. CaMK1a is a known target of Ca^2+^ signalling and several studies have established a role for Ca^2+^ signalling in regenerative growth processes in nerves. It is indeed necessary for the process of axolemmal sealing of the cut nerve process ([57]–[60] for review). Soon after transection, the cut processes of peripheral nerves re-form a growth cone and elongate. Global growth cone calcium signals can regulate cytoskeletal elements and membrane dynamics to control elongation [9]–[11]. Calmodulin (CaM) is a major Calcium binding protein which is abundant in growth cones and is a key regulator of the steering machinery [11]. Many stimulatory effects of Ca^2+^ on neurite extension and growth cone dynamics occur through the activation of the downstream effectors CaMK2a and CaMK2b [61]–[63]. The CaMKK-CaMK1 pathway has also been implicated in the regulation of growth cone motility [35], neurite outgrowth [64], [65], activity-dependent growth of dendrites [66], [67] and stabilization of spines [68] in the central nervous system. These multiple functions appear to be mediated by different CaMK1 isoforms. Thus, our data revealing that *de novo* induction of CaMK1a influences the regenerative growth of DRG neurons, at least *in vitro*, fit well with the proposed functions of this family of molecules, and extend their domain of action to the peripheral nervous system. The fact that a loss of retrogradely transported neurotrophic factors is suggested to play a role in the onset of regenerative program [55], [69] and is one of the signal leading to CaMK1 induction, support the potential role of CaMK1a in the early steps of regenerative growth.
